# Instrumented Static and Dynamic Balance Assessment after Stroke Using Wii Balance Boards: Reliability and Association with Clinical Tests

**DOI:** 10.1371/journal.pone.0115282

**Published:** 2014-12-26

**Authors:** Kelly J. Bower, Jennifer L. McGinley, Kimberly J. Miller, Ross A. Clark

**Affiliations:** 1 University of Melbourne, Department of Physiotherapy, Melbourne, VIC, Australia; 2 Royal Melbourne Hospital – Royal Park Campus, Department of Physiotherapy, Melbourne, VIC, Australia; 3 University of British Columbia, Department of Physical Therapy, Vancouver, BC, Canada; 4 Australian Catholic University, School of Exercise Science, Melbourne, VIC, Australia; University of South Florida, United States of America

## Abstract

**Background and Objectives:**

The Wii Balance Board (WBB) is a globally accessible device that shows promise as a clinically useful balance assessment tool. Although the WBB has been found to be comparable to a laboratory-grade force platform for obtaining centre of pressure data, it has not been comprehensively studied in clinical populations. The aim of this study was to investigate the measurement properties of tests utilising the WBB in people after stroke.

**Methods:**

Thirty individuals who were more than three months post-stroke and able to stand unsupported were recruited from a single outpatient rehabilitation facility. Participants performed standardised assessments incorporating the WBB and customised software (static stance with eyes open and closed, static weight-bearing asymmetry, dynamic mediolateral weight shifting and dynamic sit-to-stand) in addition to commonly employed clinical tests (10 Metre Walk Test, Timed Up and Go, Step Test and Functional Reach) on two testing occasions one week apart. Test-retest reliability and construct validity of the WBB tests were investigated.

**Results:**

All WBB-based outcomes were found to be highly reliable between testing occasions (ICC  = 0.82 to 0.98). Correlations were poor to moderate between WBB variables and clinical tests, with the strongest associations observed between task-related activities, such as WBB mediolateral weight shifting and the Step Test.

**Conclusions:**

The WBB, used with customised software, is a reliable and potentially useful tool for the assessment of balance and weight-bearing asymmetry following stroke. Future research is recommended to further investigate validity and responsiveness.

## Introduction

Impaired balance is common and a major factor influencing independence and quality of life after stroke [1]. As individuals with stroke are more likely to fall and injure themselves than the general population [2], interventions to address balance dysfunction are a strong focus in rehabilitation. Accurate, reliable and valid balance assessment tools are essential for outcome measurement in stroke-related research and clinical practice.

Instrumented tools can provide additional important information about balance performance following stroke which may not be obtained from clinical tests. Although many clinical tests are relatively quick and easy to perform they can lack sensitivity, and provide little objective information about balance strategies and movement quality; factors which are important for treatment guidance and targeted reassessment [3], [4]. Instrumented tools, such as force platforms, can be used to quantify postural sway and weight-bearing asymmetry during quiet standing [5]–[7] and dynamic activities following stroke [8]–[11]. There is potential to evaluate change over time or in response to interventions as these technologies have been found to possess good to excellent reliability for the assessment of static stance [12]–[14], weight-bearing asymmetry [11], weight-shifting activities [11]–[13], and sit-to-stand following stroke [11]. Furthermore, they may be sensitive to important attributes of balance control, as individuals who fall following stroke have been found to demonstrate larger centre of pressure (COP) sway during static stance and sit-to-stand than non-fallers [8], [15].

Force platform technologies have the potential to provide associated but unique information regarding balance control after stroke. Force platform assessment may augment clinical balance tests by providing quantitative information on postural sway, weight-bearing asymmetry and weight-shift control during balance activities. This could assist in the identification of balance deficits and falls risk, and be used to objectively monitor change over time [4], [15], [16]. Previous literature has found moderate correlations between COP variables in static stance and the Timed Up and Go [16] as well as the Berg Balance Scale [16]–[19] in individuals after stroke; however, COP velocity was found to be more strongly correlated to the static Berg Balance Scale test items [17], [19]. It has been suggested that dynamic force platform tests may correlate better with dynamic clinical balance tests [13]. For example, asymmetry of force development during sit-to-stand has been associated with reduced gait velocity, reduced cadence, increased step length asymmetry and increased single leg support time [20]. Despite the potential utility of instrumented balance assessments a key limitation is the expense and lack of accessibility for clinical settings [3]. This has severely restricted the uptake of this technology, and consequently there is a paucity of high-quality research using this technology in stroke populations.

The Wii Balance Board (WBB) (Nintendo, Kyoto, Japan) resembles a typical force platform and shows promise as a clinically useful balance assessment tool [21]. This device is relatively inexpensive and highly accessible, therefore potentially offering a more efficient method of conducting instrumented balance assessments in busy clinical settings. Researchers have begun to investigate the WBB, used with customised software (LabVIEW, National Instruments, Austin, TX, USA) that communicates with the WBB via Bluetooth, to obtain information on force development and movements in COP. The WBB has demonstrated excellent concurrent validity with laboratory-grade force platforms for quantifying COP during static standing in healthy adults (ICC  = 0.77 to >0.99) [21]–[24] and people with Parkinson's disease (ICC  = 0.92 to 0.98) [25]. Good to excellent reliability for assessment of static standing balance (ICC  = 0.64 to 0.91) [21], [23], [24] and weight-bearing asymmetry during a dynamic squatting task (ICC  = 0.75 to 0.91) [26] has been found in healthy adults. The measurement properties of WBB-based assessments have not been examined in individuals after stroke.

The primary aim of this study was to investigate the test-retest reliability of WBB-derived standing balance outcome measures in people after stroke. The secondary aim was to examine the association of these outcomes with commonly used clinical tests of dynamic balance. This novel study evaluates a low-cost protocol in a clinical setting, rather than a commercial force platform in a laboratory-only assessment. It was hypothesised that: (1) all WBB-derived variables would possess excellent test-retest reliability (ICC >0.75), (2) dynamic WBB variables would correlate more strongly with clinical tests of dynamic balance than static WBB variables, and (3) better performance on the WBB assessments would be associated with improved performance on the clinical tests.

## Methods

Thirty participants with stroke were consecutively recruited from an outpatient rehabilitation service in Melbourne, Australia. The inclusion criteria were: (1) 18 years and over, (2) non-cerebellar stroke more than three months prior, (3) able to stand for a minimum of 30 seconds unsupported, and (4) attending physiotherapy for balance or mobility issues. Exclusion criteria included: (1) medically unstable or other medical condition that could confound the results (e.g. severe arthritis or progressive neurological disorders), and (2) severe dysphasia, dyspraxia or cognitive impairment (Mini-Mental State Examination <20).

An *a priori* sample size calculation was undertaken using Stata10 (StataCorp, TX, USA). The results indicated that 30 participants would provide 80% power to detect an expected ICC of 0.80 (95% CI of 0.61–0.92), based on a previous study investigating WBB reliability in healthy adults [21]. The study was approved by the Melbourne Health Human Research Ethics Committee (2011.104) and the University of Melbourne Human Research Ethics Committee (1237303.1). Written informed consent was obtained from all participants. The results have been reported in accordance with COSMIN criteria [27].

Participants attended two testing sessions, one week apart. This was considered an appropriate length of time to reduce both test performance recall and the likelihood of change in balance. All testing sessions were conducted by a single physiotherapist and research assistant. Scores obtained from the initial testing session were not available during the second session and WBB-derived data were not displayed during the sessions. A standardised protocol was performed in the same order during each session. The order chosen represented a clinically practical testing sequence with a transition from less challenging to more demanding tasks to optimise completion of all or as many of the tests as possible. At the beginning of the second session, participants rated self-perceived change in balance performance on a 3-point global rating of change scale [28] (rating balance as the same, better or worse than the previous session), and reported any changes associated with injury, illness or medication since the first session. These questions were asked to detect self-perceived changes in balance between testing sessions. Additionally, participants were asked to rate their perceived exertion after each session using the Borg scale (rated 6 to 20) [29].

Four commonly used clinical tests of dynamic balance and mobility, with established reliability and validity in stroke [30], were administered. These tests were chosen as potentially sensitive outcomes, which approximate the balance demands of the WBB-based assessments. These were: 10 Metre Walk Test (10MWT) [31], Timed Up and Go [32], Step Test [33], and Functional Reach [34]. Participants completed the 10 MWT and Timed Up and Go in shoes and used their usual gait aids where necessary. For all tests two trials were performed and the second trial was used for analysis.

The WBBs were connected wirelessly via Bluetooth to a laptop computer running custom-written software (LabVIEW 8.5). Board calibration was performed by placing a number of known loads at varying positions on the WBB. The COP coordinates were sampled at 40 Hz and low-pass filtered at 12 Hz using an eighth-order Butterworth filter to eliminate noise [21].

The WBB-based tests consisted of: (1) static standing for 30s with eyes open and eyes closed, with both feet on one WBB; (2) static weight-bearing asymmetry, measured for 30s during standing with one WBB under each foot; (3) dynamic sit-to-stand, assessed while standing up from sitting with one WBB under each foot; and (4) dynamic mediolateral weight shifting (MLWS), which measured repeated shifting of body weight to follow a visual feedback target for 30s, using one WBB under each foot and a protocol similar to that described previously [35]. One WBB was used for static standing with eyes open and eyes closed to obtain the output variables of total, anteroposterior and mediolateral COP velocity. COP velocity was chosen as previous literature suggests it may be more reliable than other COP variables such as excursion and sway area [14], [36]. Two WBBs were used to obtain force asymmetry during the weight-bearing asymmetry assessment. Dynamic balance outcome variables during sit-to-stand included peak force and rate of force development through the lower limbs, and peak force and rate of force asymmetry between the lower limbs. These were chosen based on previous studies demonstrating reliability and validity after stroke [8], [11], [20]. The number of successful weight shifts was derived for the dynamic MLWS test. This outcome variable has previously been shown to be responsive to change after stroke [35]. The median of three trials was used for all tests except weight-bearing asymmetry, for which one trial was performed to reduce participant burden. Two practice trials were provided for the MLWS test prior to three recorded trials. Further details on these tests can be found in S1 File.

### Statistical analysis

Descriptive statistics were used to summarise baseline data and distribution was examined through Shapiro-Wilk tests and inspection of histograms. Test-retest reliability was evaluated using intraclass correlation coefficients (ICC_2,k_) with 95% CIs. Standard error of measurement (SEM) and minimum detectable change (MDC) scores were calculated and Bland-Altman plots used to further evaluate agreement [37]. Correlations between the WBB-derived outcomes and the clinical tests were assessed using Spearman's rho coefficients. Statistical analyses were undertaken with SPSS for Windows, version 21.0 (SPSS Inc, Chicago, IL) with a significance level of 0.05. Test-retest reliability strength was classified as excellent (>0.75), fair to good (0.4 to 0.74) or poor (<0.4) [38]. Correlation strength for validity was defined as excellent (0.75 to 1), moderate (0.5 to 0.74), fair (0.25 to 0.49) or poor (0 to 0.24) [39].

## Results

Thirty of 65 screened individuals screened were recruited. All participants attended both assessment sessions. Participants were a mean (SD) age of 68.3 (15.1) years, and time since stroke was a median (IQR) of 13.5 (5–45) months (Table 1). All participants were able to walk independently with or without aids. Clinical test performance scores are presented in Table 2.

**Table 1:** Participant characteristics.

Demographics	Results
Age, mean (SD), years	68.3 (15.1)
Male gender, n (%)	21 (70%)
Height, mean (SD), cm	166.7 (9.4)
Body mass, mean (SD), kg	72.5 (11.9)
Mini-Mental State Examination, median (IQR),/30^a^	28.0 (24.0–29.0)
*Stroke details*	
Right-sided lesion, n (%)	19 (63.3%)
Stroke type, infarct, n (%)	22 (73.3%)
Time since stroke, median (IQR), months^a^	13.5 (5–45)
*Comorbidities*	
Hypertension, n (%)	21 (70%)
Ischaemic heart disease, n (%)	8 (26.7%)
Diabetes, n (%)	8 (26.7%)
Dyslipidaemia, n (%)	14 (46.7%)
Atrial Fibrillation, n (%)	5 (16.7%)
Smoking history, n (%)	8 (26.7%)
Previous stroke, n (%)	2 (6.7%)

a Median and interquartile range presented where data found to be non-normally distributed.

**Table 2:** Test-retest reliability for WBB tests and clinical measures.

	Day 1 Mean (SD)	Day 2 Mean (SD)	Difference Mean (95% CI)	ICC (95% CI)	SEM	MDC (%)
*WBB-based tests*						
Eyes Open^a^						
Total COP velocity, cm/s	1.29 (0.53)	1.32 (0.65)	0.03 (−0.18, 0.24)	0.87 (0.73, 0.94)	0.19	0.53 (40.8%)
ML COP velocity, cm/s	0.50 (0.19)	0.52 (0.23)	0.02 (−0.09, 0.13)	0.87 (0.72, 0.94)	0.07	0.19 (38.0%)
AP COP velocity, cm/s	1.08 (0.47)	1.11 (0.59)	0.03 (−0.25, 0.31)	0.87 (0.73, 0.94)	0.17	0.47 (42.7%)
Eyes Closed^a^						
Total COP velocity, cm/s	2.06 (1.05)	1.98 (0.90)	−0.08 (−0.59, 0.43)	0.94 (0.87, 0.97)	0.26	0.71 (35.1%)
ML COP velocity, cm/s	0.74 (0.44)	0.70 (0.34)	−0.04 (−0.24, 0.16)	0.93 (0.86, 0.97)	0.12	0.32 (44.4%)
AP COP velocity, cm/s	1.79 (0.92)	1.72 (0.80)	−0.07 (−0.52, 0.38)	0.94 (0.87, 0.97)	0.23	0.62 (35.2%)
WBA, %BW (aff)^b^	46.3 (8.5)	45.4 (8.9)	−0.90 (−5.65, 3.85)	0.82 (0.64, 0.91)	3.61	10.00 (21.8%)
STS^c^						
Peak force (aff), %BW*	54.3 (7.0)	52.7 (6.2)	1.60 (−2.33, 5.53)	0.90 (0.76, 0.96)	2.21	6.14 (11.5%)
Peak force asymmetry†	0.89 (0.21)	0.86 (0.19)	−0.03 (−0.15, 0.09)	0.96 (0.90, 0.98)	0.04	0.12 (7.7%)
Peak RFD, %BW/sec*	424.4 (124.5)	411.9 (128.0)	−12.5 (−87.54, 62.54)	0.94 (0.85, 0.97)	30.50	84.53 (20.2%)
Peak RFD asymmetry†	0.71 (0.21)	0.75 (0.23)	0.04 (−0.09, 0.17)	0.89 (0.75, 0.95)	0.07	0.19 (26.0%)
MLWS, no/30s^d^	10.0 (3.7)	10.3 (3.9)	0.30 (−1.74, 2.34)	0.98 (0.96, 0.98)	0.52	1.45 (14.3%)
*Clinical tests*						
10 Metre Walk Test, m/s	0.96 (0.34)	0.95 (0.33)	−0.01 (−0.18, 0.16)	0.97 (0.93, 0.98)	0.06	0.16 (16.7%)
Timed Up and Go, s	17.7 (10.5)	17.4 (10.4)	−0.30 (−5.70, 5.10)	0.99 (0.98, 0.99)	1.05	2.91 (16.6%)
Step Test (aff), no/15s	9.2 (4.9)	9.6 (5.0)	0.40 (−2.16, 2.96)	0.98 (0.97, 0.99)	0.69	1.92 (20.4%)
Step Test (unaff), no/15s	8.2 (4.6)	8.5 (4.6)	0.30 (−2.08, 2.68)	0.95 (0.89, 0.97)	1.03	2.85 (34.1%)
Functional Reach, cm	27.8 (7.5)	29 (7.6)	1.20 (−2.70, 5.10)	0.91 (0.81, 0.96)	2.25	6.24 (22.0%)

Abbreviations: ICC, Intraclass Correlation Coefficient; SEM, Standard Error of Measurement; MDC, Minimal Detectable Change; COP, centre of pressure; ML, mediolateral; AP, anteroposterior; WBA, weight-bearing asymmetry; BW, body weight; aff, affected lower limb; unaff, unaffected lower limb; STS, sit-to-stand; RFD, rate of force development; MLWS, mediolateral weight shifting.

*Calculated relative to body mass;

† Calculated as affected/unaffected lower limb;

a n = 30;

b n = 27;

c n = 23;

d n = 28.

No participant reported change on the 3-point global rating of change scale. Scores between the two testing sessions did not significantly differ for any of the WBB variables or clinical tests (Table 2). Two participants were unable to perform the sit-to-stand test. Furthermore, additional data points were missing for the weight-bearing asymmetry, sit-to-stand and MLWS tests due to testing time constraints (Table 2). This was related to participants' time limitations due to attendance of concurrent therapy appointments. The total session length varied from 45 to 90 minutes, with an average of 64.8 minutes. The combined WBB assessments took an average of 21.0 (6.3) minutes to complete. Over the 60 testing sessions, the median (IQR) Borg rating was 10 (9–11), or ‘fairly to very light’ self-perceived exertion.

Test-retest reliability was excellent for all WBB variables (ICC  = 0.82 to 0.98; Table 2). MDC scores ranged from 7.7% to 44.4% with larger scores observed for the static WBB variables. Bland-Altman plots for WBB variables are presented in S2 File, showing no indication of systematic bias or trends between sessions.

WBB variables highly correlated with each other (r_s_≥0.75) were deemed redundant based on a theoretical assumption of similarity (S3 File). Clinical judgement was then used to prioritise these similar outcome variables according to their relevancy to balance performance and select the five key outcome variables for correlation with the clinical tests (Table 3). Apart from the Functional Reach (*p*>0.05), correlations of fair to moderate strength were found between performance on clinical balance tests and dynamic MLWS performance (r_s_ = 0.47 to −0.57), with a greater number of successful shifts indicative of better performance on the clinical tests. Relationships between clinical test performance and total COP velocity during the static eyes open task were found to be fair in strength, with the exception of Functional Reach, which was moderately correlated (r_s_ = −0.61). These associations were all in the direction of increased sway being related to worse performance on the clinical tests. No correlations were found between the clinical balance tests and static weight-bearing asymmetry or dynamic sit-to-stand force variables (*p*>0.05).

**Table 3:** Correlations between WBB tests and clinical tests (Spearman's rho).

	10 MWT	Timed Up and Go	Step Test (aff)	Functional Reach
EO total COP velocity^a^	−0.44*	0.44*	−0.41*	−0.61**
WBA^b^	0.10	−0.13	0.14	−0.13
STS Peak force asymmetry^c^	0.04	−0.03	0.18	−0.35
STS Peak RFD^c^	0.08	−0.23	0.06	0.27
MLWS^d^	0.47*	−0.57**	0.53**	0.44

Abbreviations: 10 MWT, 10 Metre Walk Test; aff, affected lower limb in stance; EO, eyes open; COP, centre of pressure; WBA, weight-bearing asymmetry; STS, sit-to-stand; RFD, rate of force development; MLWS, mediolateral weight shifting.

* Significant at *p*<0.05; ** Significant at *p*<0.01; ^a^n = 30;

b n = 27;

c n = 25;

d n = 28.

## Discussion

The results of this study suggest that the WBB, used with customised software, can be a reliable and potentially useful tool for the assessment of static and dynamic standing balance following stroke. Test-retest reliability was found to be consistently excellent across all WBB-derived variables, and the MDC values presented in this paper may assist clinicians in detecting change in balance performance over time or in response to treatment interventions in individuals with stroke. The stronger correlations found between several outcomes (e.g. WBB-derived weight shifting ability and the Step Test) highlight task-specific associations and support the validity of these tests. The lower correlations found between several outcomes (e.g. sit-to-stand and gait) may reflect the different aspects of balance performance being assessed or the influence of factors other than balance impacting on performance in these tests. The low strength correlations may also mean that the WBB-derived outcomes are not relevant for assessing some aspects of balance. However, this study indicates that a WBB-based assessment can provide additional information on balance, such as postural sway, asymmetry and control of weight-shift ability, which may be used to enhance balance testing in research and clinical practice.

The excellent test-retest reliability found for the static WBB tests was consistent with prior studies using laboratory-based force platform technologies in stroke [11], [12], [14] and the WBB in healthy individuals [21]. Despite high ICC values, the MDC scores for static WBB tests were larger than those previously found in healthy adults (MDC  = 23.9 to 27.9%) [21], [23]. This was likely due to the greater variation between individuals in the present study. SEM scores from static balance COP velocity variables were of a similar magnitude to a recent study in stroke (SEM  = 17.5 to 18.9%) [14]. Therefore, although the WBB can be reliably used to assess static standing balance following stroke, relatively large change scores may be needed to be confident that real change has occurred.

The dynamic WBB tests were also found to be highly reliable, with lower MDC scores than the static tests. Consistent with previous research [11], the reliability of force production during sit-to-stand after stroke was high. Dynamic weight shifting tests using diverse force platform-based protocols have been previously examined in stroke with good to excellent reliability outcomes [11]–[13]. The reliability of the MLWS test used in the current study was found to be excellent. Demonstrated improvement in the MLWS during stroke rehabilitation [35], [40] and the relatively small MDC score found in the current study supports the potential clinical utility of this outcome. Although it is imperative that assessment tools possess adequate reliability, other properties such as validity and responsiveness are clinically important.

Our hypothesis that the dynamic WBB-derived variables would correlate more strongly with clinical balance tests than the static variables was not clearly supported; however, there was some evidence of task-related associations. Although the dynamic MLWS test demonstrated significant moderate correlations with three of the four clinical tests, COP velocity in static standing was also found to have associations of comparable strength. Interestingly, the MLWS correlated more strongly with those tests requiring similar task demands of lateral weight transference (i.e. Timed Up and Go and Step Test). However, correlation with the 10 MWT was not as strong, perhaps reflecting other contributions, such as muscle strength, to this test which is more an assessment of mobility than balance. The additional cognitive and perceptual challenge required to perform the MLWS test may have influenced the strength of association with clinical tests. The MLWS test requires the individual to respond to changing visual and auditory cues while controlling weight-shift in a coronal plane. This test could present a potential means of collecting unique information about balance performance, including the ability to use real-time visual cues as feedback during a balance task. Conversely, COP velocity in static standing correlated most strongly with the Functional Reach. This may be explained by the similarity in demand for COP stability in holding a forward reach position. Higher correlations may have been observed between the MLWS test and a lateral reach test, or by comparing Functional Reach performance to a dynamic WBB test assessing movement in the sagittal plane. Prior research has found similarly low correlations between static COP sway variables and gait performance [5], [13]; however, moderately strong associations between COP sway and clinical tests including the Berg Balance Scale [16]–[19] and Timed Up and Go have previously been demonstrated [16].

Static weight-bearing asymmetry and sit-to-stand WBB variables were found to have surprisingly low correlations with the clinical tests. It has been suggested that dynamic force platform-derived assessments, such as weight shifting and sit-to-stand, are more likely to correlate with dynamic clinical balance tasks [11], [13]; however, static weight-bearing asymmetry after stroke has been previously linked to gait performance [5]. The low strength associations found in our study may have been influenced by the comparatively small magnitude of asymmetry found in our participant group. Furthermore, weight-bearing asymmetry is affected by factors other than balance, such as strength, somatosensation, hemi-inattention and perception of verticality [7]. Similarly, although sit-to-stand ability has been linked to balance outcomes [8], [9], lower limb strength rather than balance may have a stronger influence on performance [10]. Higher correlations may have been found with alternate clinical measures such as a timed sit-to-stand test. Sit-to-stand outcomes in this study may also have been influenced by the sample having relatively mild post-stroke impairments. Evidence supporting the clinical importance of sit-to-stand performance [10], [20] highlights the potential usefulness of this outcome, despite the low correlations found in this study.

This study had several limitations. Although participants with a range of post-stroke deficits were consecutively recruited, generalisability may be limited due to enrolment from a single facility of individuals with adequate function to participate in the testing protocol. Despite using standardised testing protocols, variation in scores may have been affected by learning, fatigue, motivation, difficulties in obtaining correct foot placement, comprehension of instructions and assessor performance. Inter-rater reliability was not assessed in this study; however, the objective nature of the assessment tool reduces the likelihood of variability both within and across examiners. Although all participants completed the static balance testing, data were lost from up to seven participants on other tests due to inability to physically perform the tests or time constraints. Participants with missing data tended to be those who were lower functioning, therefore potentially reducing the heterogeneity of the data and attenuating the strength of associations. The WBB tests were not validated against another force platform in this study; however, previous research has demonstrated high concurrent validity in healthy populations and in neurological populations with impaired balance. This study only employed a small number of clinical tests and stronger correlations may have been found with the selection of additional outcomes more closely related to the WBB-derived measures such as static balance and timed sit-to-stand testing. Furthermore, using other validated scales of balance, such as the Berg Balance Scale, Postural Assessment Scale for Stroke, or Community Balance and Mobility Scale, may have resulted in different findings. Finally, the customised software developed for this study is at present not widely available, and limits widespread clinical utility. However, the WBB is easily programmable with example code available freely on the internet.

## Conclusions

To the authors' knowledge, this study is the first to examine measurement properties of the WBB, used with customised software, for assessment of balance after stroke. Instrumented assessment of static and dynamic balance using the WBBs was found to be highly reliable. Correlations between WBB and clinical tests varied from no correlation to moderate at best, with some evidence of task-specific relationships. This tool may be used to strengthen outcome assessment for research and practice; however, it is recommended that future research be conducted to further explore clinically important aspects of validity and responsiveness to change over time.

## Supporting Information

S1 File **Description of Wii Balance Board tests.**
(PDF)

S2 File **Bland-Altman plots.**
(PDF)

S3 File **Correlations between Wii Balance Board variables.**
(PDF)

S4 File **Individual study outcome data.**
(XLSX)
