# Supplementary material for: Instrumented Static and Dynamic Balance Assessment after Stroke Using Wii Balance Boards: Reliability and Association with Clinical Tests
Source: PLoS One. 2014 Dec 26;9(12):e115282. doi: 10.1371/journal.pone.0115282 (PMC4277284; doi:10.1371/journal.pone.0115282)
Supplement: S1 File — Description of Wii Balance Board tests. (PDF) [file pone.0115282.s001.pdf]

File S1. Description of Wii Balance Board tests

**Eyes open and eyes closed**	**Weight-bearing asymmetry**
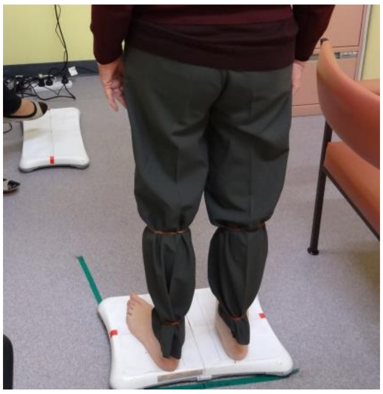	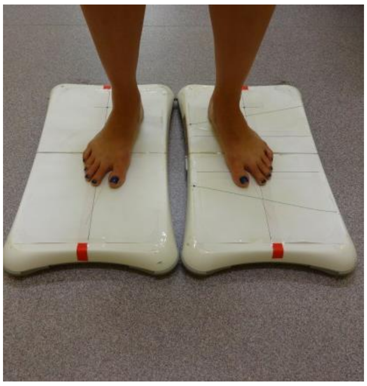
Description: Participant stands on single Wii Balance Board. Heels 17cm apart and toe-out angle of 14°. Asked to “stand as still as you can” focusing on target on wall in front. Eyes closed as per eyes open but with closed eyes.*<br>Duration: 30 seconds x 3 trials<br>Outcome variables: Total centre of pressure (COP) velocity, cm/s; mediolateral COP velocity, cm/s; anteroposterior COP velocity, cm/s	Description: Participant stands on two Wii Balance Boards. Foot position and instructions as per eyes open.*<br>Duration: 30 seconds x 1 trial<br>Outcome variables: % body weight on affected lower limb
**Sit-to-stand**	**Mediolateral weight shifting**
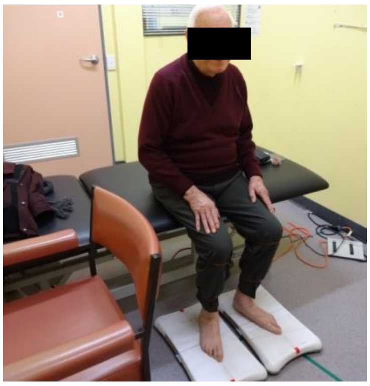	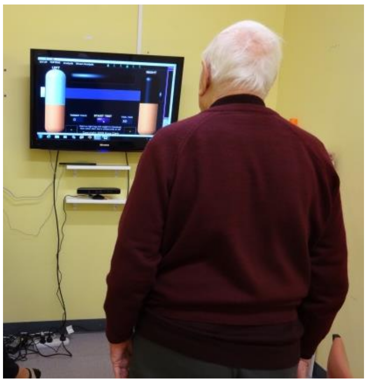
Description: Participant sits with feet on two Wii Balance Boards. Heels 17cm apart and toe-out angle of 14°. Seat positioned to achieve 75° knee flexion and 5-10° ankle dorsiflexion. Participants were asked to stand without using their upper limbs for support.*<br>Duration: Self-selected pace x 3 trials<br>Outcome variables: Peak force (affected lower limb),% body weight; Peak force asymmetry (affected / unaffected lower limb); Peak rate of force development (RFD) (affected lower limb), % body weight /sec; Peak RFD asymmetry (affected / unaffected lower limb)	Description: Participant stands on two Wii Balance Boards. Heels 34cm apart and toe-out angle of 14°. Guided by visual feedback on a television screen, to shift body weight side-to-side to hold for one second in a target area (representing 60-80% of body weight).*<br>Duration: 30 seconds x 5 trials<br>Outcome variables: Number of shifts in 30 seconds

* A chair was placed nearby on the less affected side in case of loss of balance. Trials were deemed unsuccessful if the participant touched the chair, or if physical assistance was required for steadying.
