# Supplementary material for: Instrumented Static and Dynamic Balance Assessment after Stroke Using Wii Balance Boards: Reliability and Association with Clinical Tests
Source: PLoS One. 2014 Dec 26;9(12):e115282. doi: 10.1371/journal.pone.0115282 (PMC4277284; doi:10.1371/journal.pone.0115282)
Supplement: S2 File — Bland-Altman plots. (PDF) [file pone.0115282.s002.pdf]

File S2. Bland-Altman plots

A. Eyes open total COP velocity

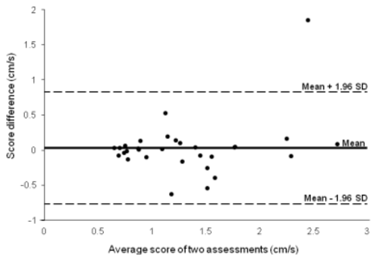

B. Eyes open ML COP velocity

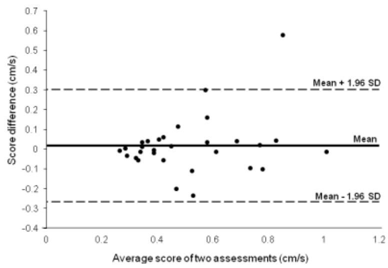

C. Eyes open AP COP velocity

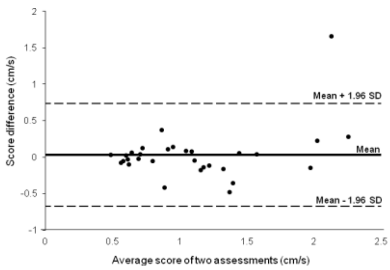

D. Eyes closed total COP velocity

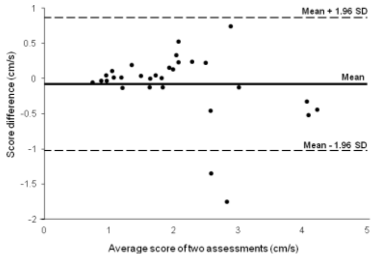

E. Eyes closed ML COP velocity

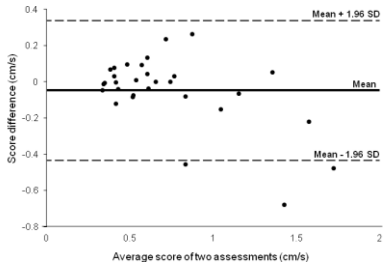

F. Eyes closed AP COP velocity

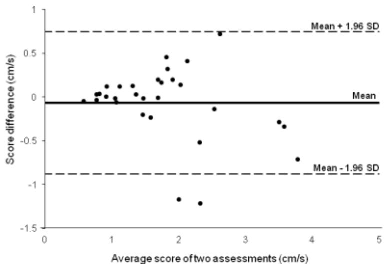

G. Weight-bearing asymmetry†

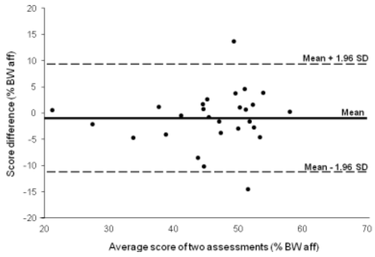

H. STS peak force (affected)* ‡

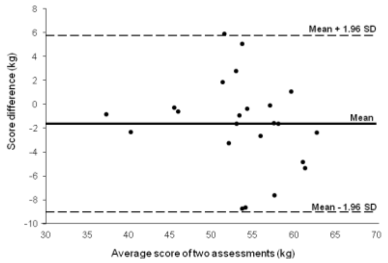

I. STS peak force asymmetry‡

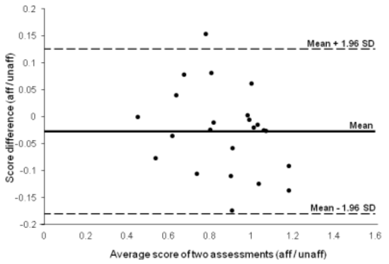

J. STS peak RFD* ‡

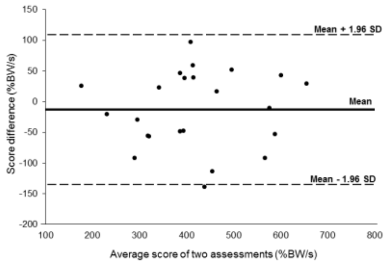

K. STS peak RFD asymmetry‡

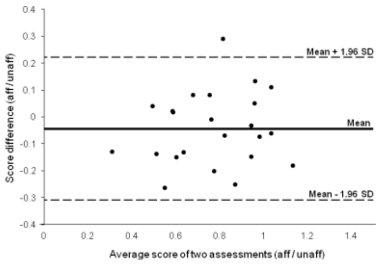

L. Mediolateral weight shifting§

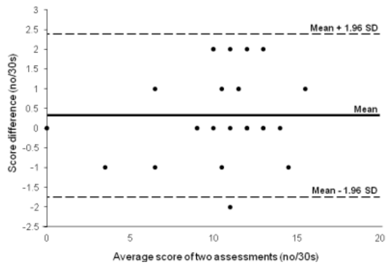

Abbreviations: COP, centre of pressure; ML, mediolateral; AP, anteroposterior; STS, sit-to-stand; RFD, rate of force development; BW, body weight; aff, affected lower limb; unaff, unaffected lower limb

* Calculated relative to body mass; † n=27; ‡ n=23; § n= 28
