# Supplementary material for: Instrumented Static and Dynamic Balance Assessment after Stroke Using Wii Balance Boards: Reliability and Association with Clinical Tests
Source: PLoS One. 2014 Dec 26;9(12):e115282. doi: 10.1371/journal.pone.0115282 (PMC4277284; doi:10.1371/journal.pone.0115282)
Supplement: S3 File — Correlations between Wii Balance Board variables. (PDF) [file pone.0115282.s003.pdf]

File S3. Correlations between Wii Balance Board variables (Spearman's rho)

	EO total vCOP	EO ML vCOP	EO AP vCOP	EC total vCOP	EC ML vCOP	EC AP vCOP	WBA (%BW aff)†	MLWS (no/30s)§	STS peak force (aff)‡‖	STS peak asymmetry‡ #	STS RFD (%BW/s) ‡‖	STS RFD asymmetry‡ #
EO total vCOP	1											
EO ML vCOP	0.908**	1										
EO AP vCOP	0.992**	0.887**	1									
EC total vCOP	0.910**	0.885*	0.891**	1								
EC ML vCOP	0.848**	0.929**	0.811**	0.918**	1							
EC AP vCOP	0.915**	0.846**	0.904	0.988**	0.867**	1						
WBA (%BW aff)†	-0.134	-0.235	-0.123	-0.225	-0.318	-0.192	1					
MLWS (no/30s)§	-0.338	-0.341	-0.320	-0.433*	-0.388	-0.460*	0.240	1				
STS peak force (aff) ‡‖	0.465*	0.410*	0.481*	0.318	0.239	0.323	0.400*	0.128	1			
STS peak asymmetry‡ #	0.253	0.199	0.280	0.043	-0.036	0.053	0.549**	0.242	-0.865**	1		
STS RFD (%BW/sec) ‡‖	-0.003	-0.036	0.004	-0.022	0.033	-0.041	-0.327	0.149	-0.183	-0.302	1	
STS RFD asymmetry‡ #	0.261	0.074	0.293	0.072	-0.062	0.097	-0.530**	0.243	0.713**	-0.831**	-0.105	1

Abbreviations: EO, eyes open; EC, eyes closed; vCOP, centre of pressure velocity (cm/s); ML, mediolateral; AP, anteroposterior; WBA, weight bearing asymmetry; BW, body weight; aff, affected lower limb; STS, sit-to-stand; RFD, peak rate of force development; MLWS, mediolateral weight shifting

* Significant at $P < 0.05$; ** Significant at $P < 0.01$; † n=27; ‡ n=25; § n=28; ‖ Calculated relative to body mass; # Calculated as affected / unaffected lower limb
